# Screening *Actinomycetes* extracts for antimicrobial compounds against methicillin-resistant *Staphylococcus aureus* and helper-compounds against aminoglycoside-resistant *E. coli*

**DOI:** 10.12688/openreseurope.19988.2

**Published:** 2025-08-11

**Authors:** Dina Al Nahhas, Sandra Marina Wellner, Margherita Sosio, Sonia I Maffioli, Salvatore Pisanu, Sergio Uzzau, Daniela Pagnozzi, Stefano Donadio, John Elmerdahl Olsen

**Affiliations:** 1 Porto Conte Ricerche, Alghero, 07041, Italy; 2 Veterinary and Animal Sciences, University of Copenhagen, Frederiksberg, 1870, Denmark; 3 Naicons, Milano, 20139, Italy; 4 Biomedical Sciences, University of Sassari, Sassari, 07100, Italy

**Keywords:** Antimicrobial screening, natural products, purification, aminoglycosides, MRSA, Escherichia coli

## Abstract

**Background:**

Innovative antibiotic discovery strategies are urgently needed to successfully combat infections caused by multi-drug-resistant bacteria.

**Methods:**

We employed a direct screening approach to identify compounds with antimicrobial and antimicrobial helper-drug activity against Gram-positive and Gram-negative bacteria. We used this platform in two different strains of methicillin-resistant *Staphylococcus aureus* (MRSA) and aminoglycoside-resistant strains of *Escherichia coli* to screen for antimicrobials compounds, which potentiate the activity of aminoglycoside antibiotics. Screening was performed with 75 known microbial products and 880 extracts from *Actinomycetes* from a collection at the company Naicons.

**Results:**

The antibiotics rifamycin O and thermorubin inhibited the growth of neomycin-resistant *E. coli* in combination with 1/8 MIC of neomycin, suggesting a potential application as adjuvant drugs for neomycin. Additionally, in the *Actinomycetes* extract screen, one extract with antimicrobial activity and one extract with gentamicin adjuvant activity against gentamicin-resistant *E. coli* were identified, demonstrating the applicability of the screening approach. Against MRSA, the paramagnetoquinones, the lantibiotic NAI-107 and the spirotetronate NAI-414 showed the most pronounced antimicrobial activity. Difference is susceptibility towards antimicrobials and extracts were observed between the two MRSA strains used for screening.

**Conclusion:**

Compounds with antibacterial and helper drug activity were identified using our screening approach. The results demonstrate the importance of strain selection in antimicrobial screening and highlight the potential of natural products as a source of potential new antibacterial and adjuvant therapies against both Gram-positive and Gram-negative bacteria.

## Plain Language Summary

Every year, nearly 700,000 people worldwide die from infections that no longer respond to antibiotics. With drug resistance on the rise, there is an urgent need for novel strategies to fight these deadly bacteria.

In our study, we developed a laboratory screening method to find new antibacterial substances and helper compounds that can boost the effect of existing antibiotics. We focused on two major challenges: methicillin-resistant *Staphylococcus aureus* (MRSA), which causes severe infections, and strains of *Escherichia coli* ( *E. coli*) that resist aminoglycoside antibiotics.

Our screening approach identified several promising candidates: The antibiotics rifamycin O and thermorubin, in combination with a low dose of neomycin, were effective against neomycin resistant *E. coli*, suggesting these compounds could serve as helper-drugs for neomycin resistant variant of this species. Further, a screen among natural extracts identified one extract with direct antimicrobial activity against *E. coli* and another that enhanced the activity of gentamicin against *E. coli* resistant to this antibiotic. For MRSA, several compounds including paramagnetoquinones, the lantibiotic NAI-107, and the spirotetronate NAI-414 showed strong antibacterial effects.

With bacteria becoming resistant to many conventional antibiotics, finding new active compounds and ways to boost existing treatments is critical. Our findings highlight the potential of natural products to provide new drug options and points to combination therapies which exploits previously not recognized synergies between known antibiotics. Such approaches could lead to more effective treatments against antibiotic-resistant infections and contribute to the reduction of the worldwide death toll.

## Introduction

The increasing prevalence of antibiotic-resistant pathogens poses a major threat to human and animal health, underscoring the urgent need for novel antibiotics and alternative strategies to treat bacterial infections ^1– 3^. According to the 2024 WHO report on AMR, 50% of the fatal burden associated with antimicrobial resistance (AMR) is linked to two pathogens: *S. aureus* and *E. coli* ^4^. With *E. coli* classified as a “critical” priority and methicillin-resistant *S. aureus* (MRSA) as “high” priority ^5, 6^, both pathogens remain central targets for antimicrobial research, where the development of both novel antibiotics and adjuvant therapies could help to overcome current treatment limitations.

Adjuvant therapies or “helper-drugs” are used in combination with antibiotics to enhance their efficacy. These compounds have typically limited or no antimicrobial activity on their own, but when used together with an antibiotic, they can significantly increase its ability to kill or inhibit bacterial growth ^7^. An ideal helper compound can penetrate the bacterial cell envelope, has a low tendency for resistance development, and specifically targets bacterial components without harming the host ^3, 8^. This approach is especially valuable for challenging pathogens, such as Gram-negative bacteria, which possess a double membrane structure with embedded efflux pumps, explaining the low rate of novel antimicrobials for this group compared to Gram-positive bacteria ^9, 10^.

The class of aminoglycosides exhibits broad-spectrum activity against Gram-positive and Gram-negative bacteria. These antibiotics bind to the 16S subunit of the bacterial ribosome, causing error-prone protein biosynthesis ^11– 13^. Aminoglycosides, such as amikacin, gentamicin (GEN), and tobramycin, are used to treat life-threatening infections caused by multi-drug-resistant Gram-negative bacteria ^13, 14^. Additionally, streptomycin (STREP) and neomycin (NEO) are often the drugs of choice for treating infections caused by *Yersinia pestis* and *Francisella tularensis*, as well as *E. coli*-related infections in animals, for example post weaning diarrhea in pigs ^13, 15– 17^.

*Staphylococcus aureus*, particularly MRSA, is a versatile pathogen capable of causing a wide range of infections, from skin infections to life-threatening conditions such as pneumonia and sepsis ^18^. MRSA strains, due to their resistance to multiple antibiotics, present significant challenges in healthcare and community settings ^19, 20^. This study focuses on two MRSA strains with distinct genomic profiles: JE2, a highly virulent community-associated MRSA (CA-MRSA) strain ^21^, and ST398, a livestock-associated MRSA (LA-MRSA) strain associated with zoonotic infections ^22^.

To identify novel antimicrobial and helper-compounds, phenotypic screens can be used ^23, 24^. Whole-cell screening, first established in the 1920s by S. Waksman and his students, involved screening soil-dwelling *Actinomycetes* for antibacterials which led to the identification of antimicrobial compounds including streptothricin and STREP ^3, 25, 26^. Whole cell screening approaches have the advantage that compounds, which are not able to reach their target in a sufficient concentration, are eliminated from the beginning, however after the identification of a hit compound, their mechanism of action must be determined ^23, 24, 27^.

*Actinomycetes*, known for their prolific production of bioactive compounds, have historically been a rich source of antibiotics and remain promising for discovering novel antimicrobial agents and adjuvant compounds ^28^. In the current study, we screened a library of 75 known antimicrobials and 880 extracts from *Actinomycetes*. To resensitize aminoglycoside-resistant *E. coli* to this class of antibiotics, we developed a helper-drug screen to identify compounds that enhance aminoglycoside activity. For MRSA strains, we screened a library of 75 known compounds and 160 *Actinomycetes* extracts for novel antimicrobials, followed by deconvolution of active molecules from promising hits.

## Methods

### Preparation of antimicrobial compound and extract plates

Two types of screening plates were prepared, a compound plate and extract plates from *Actinomycetes*. The antimicrobial compound plate contained 75 compounds with known antimicrobial activity prepared in-house. Details on compounds and their respective solvents are provided in Tables **S1 and S2**. Stock solutions of each compound (100 mg/L) were prepared in appropriate solvents, and 100 to 200 μL of each solution was dispensed into individual wells of 96-well plates. Plates were then dried overnight at mild heat to evaporate the solvent completely, leaving a dried residue of compound in each well. The dried plates were stored refrigerated at 4 °C until use. Immediately prior to screening, each well was reconstituted by adding 100 μL of 10% DMSO, followed by incubation at room temperature for approximately 30 minutes to ensure full dissolution of the compounds. This process generated assay-ready compound solutions at the desired working concentrations. Final assay concentrations were typically 20 mg/L for *E. coli* and 10 mg/L for MRSA, consistent with concentrations used in NAICONS protocols and chosen to approximate known antimicrobial activity thresholds.

For the *Actinomycetes* extract plates, dried extracts from strains in the NAICONS microbial collection ^29^ were utilized. Full extracts were prepared by culturing strains in 2 mL volumes, followed by addition of 4 mL ethanol. Cultures were shaken for 1 hour at 30°C and then centrifuged at 4000 rpm for 8 minutes to clarify. Fractionated extracts were also prepared from both mycelium and cleared broth. Mycelium extracts were obtained by treating the pellet from 8 mL cultures with sequential ethanol washes at 20%, 50%, and 100% concentrations (4 mL each), with shaking for 1 hour at 30°C and subsequent centrifugation at 4000 rpm for 8 minutes after each wash. Cleared broth extracts were treated with HP20 resin (0.8 mL), shaken for 1 hour at 30°C, then centrifuged at 8000 rpm for 1 minute. The resin was subsequently extracted with increasing ethanol concentrations (5%, 30%, and 80%), shaking for 15 minutes at 30°C followed by centrifugation at 8000 rpm for 1 minute after each step. All extracts were dried overnight at room temperature and stored at 4°C until use. Prior to screening, extracts originally prepared as 100 μL aliquots were resuspended in 100 μL of 10% DMSO. For activity testing, 5 to 20 μL of the resuspended *Actinomycetes* extracts were applied per assay well.

### Bacterial strains and cultivation of bacterial strains

Four strains of *E. coli* ^30^ were utilized in this study. The parent strain was *E. coli* K12 MG1655 ^31^, and in addition, strains, which were resistant to selected aminoglycoside antibiotics due to the presence of plasmid encoding resistance to NEO ( *aph(3’)-Ia* gene), GEN ( *aac(3)-IV* gene) or STREP ( *strAB* gene) were used (Table S3). Strains were grown in Luria Broth (LB) Lennox with shaking or on LB agar plates overnight at 37°C. When appropriate, the media was supplemented with antibiotics including 50 mg/L NEO, 50 mg/L STREP, 20 mg/L GEN or 50 mg/L Chloramphenicol (CHL) (Sigma-Aldrich, Copenhagen, Denmark).

MRSA strains consisted of JE2 ^21^ and ST398 ^22^. These were grown in Tryptic Soy Broth (TSB) with shaking overnight at 37 °C. When appropriate, 1 mL of the culture was added to 10 mL of Mueller-Hinton broth (CA-MHB) cation adjusted, mainly magnesium and calcium. TSB, LB, and MHB used are commercially available from BD ( www.bd.com/en-us/).

### Testing antimicrobial (helper-) activity of compounds

**Figure 1.**
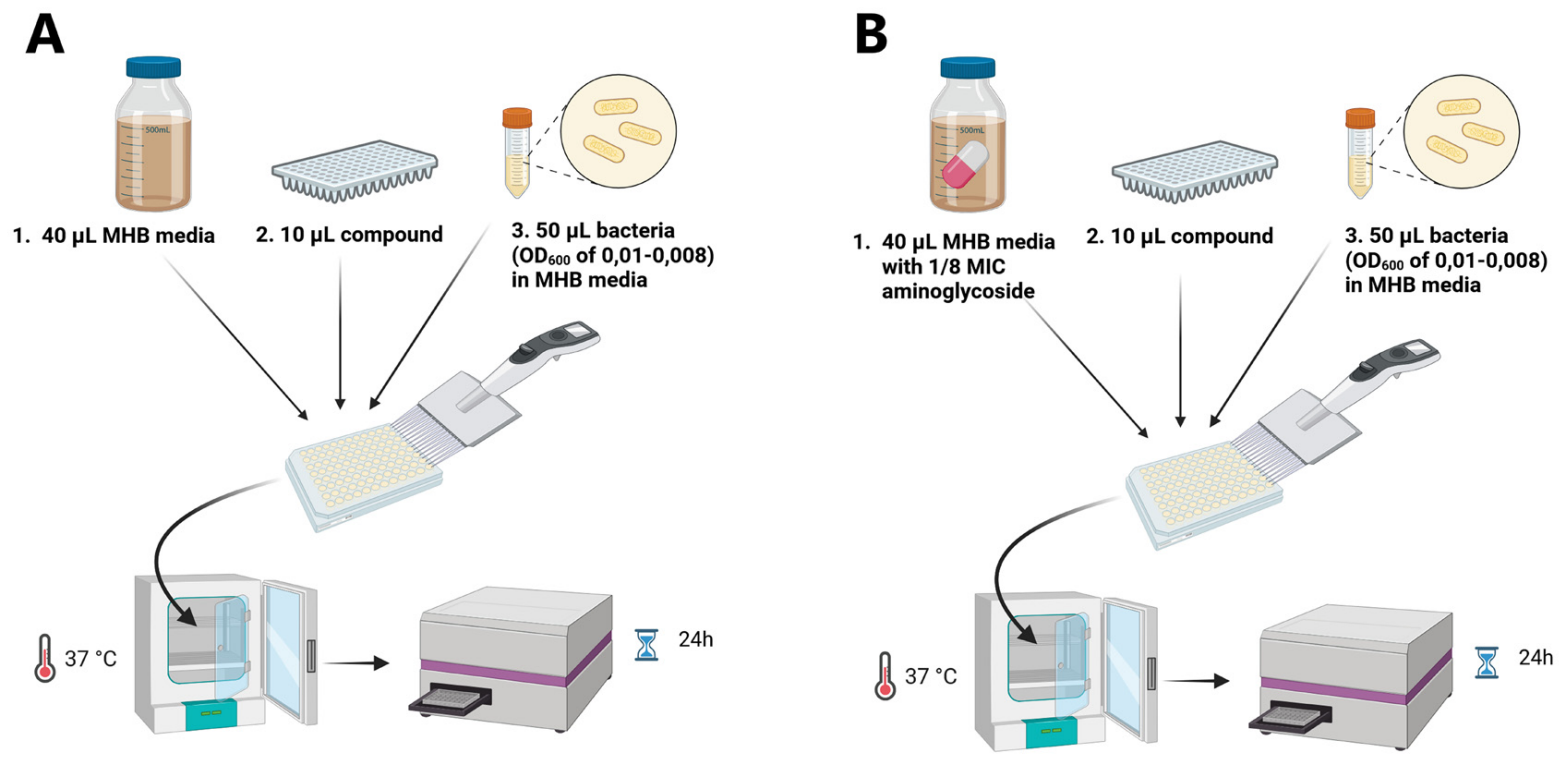
Schematic representation of the screening approach for antimicrobials ( **A**) and aminoglycoside helper-compounds ( **B**). In a 96-well plate in each well, 40 μL MHB media, 10 μL compound and 50 μL bacterial suspension were mixed, incubated at 37 °C and the OD _600_ was measured after 24h using a plate reader. This figure was created using BioRender.com.

The minimum inhibitory concentration (MIC) of each antimicrobial compound was determined against both *E. coli* and MRSA strains using the broth micro-dilution method, following CLSI guidelines ^32^. For the assay, we prepared each well with 40 μL of MHB media, 10 μL of the compound (final concentration: 20 mg/L for *E. coli* and 10 mg/L for MRSA, Table S1, S2), and 50 μL of bacterial culture ( *E. coli* or MRSA), as shown in ( Figure 1A). For testing aminoglycoside helper-compounds, a final concentration of 1/8 MIC of the relevant aminoglycoside (NEO, STREP, or GEN) was added to the MHB, along with 40 μL of MHB media, as shown in ( Figure 1B). The bacterial suspensions were prepared by adding 100 μL of aminoglycoside-resistant *E. coli/*MRSA of an optical density (OD) _600_ of 0.08-0.1 (corresponding to a McFarland standard of 0.5) to 10 mL MHB media. Afterwards, the 96-well plate was incubated at 37 °C for 24 h followed by an OD measurement using a plate reader.

### Screening of extracts and deconvolution of active molecules

To clarify the screening strategy, a total of 880 Actinomycetes-derived extracts were screened against *E. coli* to identify helper-compound activity, while a smaller subset of 160 extracts was tested against MRSA for direct antimicrobial effects. This difference reflects our experimental focus: early hit identification against MRSA allowed us to prioritize purification and characterization steps, while *E. coli* required a broader screen due to the difficulty in finding active hits.

On the testing day, each extract was prepared by suspending 100 µL in 10% DMSO. Then, 10 µL of each extract was added to wells containing 40 µL of bacterial solution and 50 µL of MHB, as outlined above. Extracts showing 70% or higher inhibition against MRSA compared to growth without the extract (calculations detailed below) were selected for verification, fractionation, and purification to identify the active compound. For *E. coli*, extracts demonstrating potential helper-drug activity, follow-up experiments were conducted by adding 5–20 μL of the extract, 45–30 μL of MHB, and 50 μL of bacterial suspension to a 96-well plate (final volume: 100 μL). Incubation and OD measurements were performed as described above.

Upon activity confirmation, six fractionated extracts (three from mycelium and three from the cleared broth) were also tested in serial dilutions. At first, the fractions were suspended in 125 µL of 10% DMSO and tested as for the full extracts. Active fractions were further sub-fractionated into 24 fractions (one fraction per minute) using High-Performance Liquid Chromatography (HPLC). We used a Shimadzu HPLC system LC 2010A-HT (Kyoto, Japan) equipped with a Merck LiChrospher RP-18 reverse-phase column (5 μm, I.D. × L 4.6 × 125 mm, Merck, Darmstadt, Germany) at 50°C. The mobile phases consisted of 0.05% formic acid (HCOOH) for phase A and acetonitrile (ACN) for phase B. The flow rate was set at 1 mL/min. Samples were fractionated using a linear gradient: ACN was increased from 10% to 95 in 18 minutes, followed by 5 minutes at 95%. UV detection was carried out at 230 and 270 nm. The 24 subfractions were dried using a speed vacuum concentrator and resuspended later in 10% DMSO for further screening, using the same screening technique as above. Upon activity confirmation, the active fraction was analyzed using high-resolution mass spectrometry (HRMS) to identify the specific compound responsible for the activity. For each active fraction detected, the adjacent inactive fractions (preceding and subsequent) were also analyzed to monitor the elution peak of the active fraction. The analysis was performed using a Vanquish UHPLC system connected to an Orbitrap Exploris 120 mass spectrometer, utilizing a YMC-Triart ODS column. The mobile phase included 0.1% formic acid in water (A), LCMS grade acetonitrile (B), and LCMS grade isopropyl alcohol (C), with a gradient flow program over a 23-minute cycle, as described in 33. The flow rate was set at 0.8 mL/min with a sample injection volume of 8 μL at 40°C. During the separation, 75% of the flow was directed to a diode array detector and 25% to the mass spectrometer, which was equipped with a heated electrospray ionization source operating in both positive and negative modes. Finally, MS/MS and UV-Vis spectra were analyzed and matched against both internal ^34^ and external databases ^35– 38^ to identify the active compounds. Finally, each sub-fraction was then suspended in the appropriate volume, and their activity against MRSA was further tested.

### Assessing bioactivity of identified molecules

To evaluate the bioactivity of the identified molecules against MRSA strains, we obtained purified elaiophylin (Santa Cruz Biotechnology, Inc.) and nigericin sodium salt (MedChemExpress.com). Each molecule was initially dissolved in DMSO to prepare a 1 mg/mL stock solution. MIC testing and checkerboard assays were then conducted in combination with ampicillin (AMP) to assess potential synergistic effects.

### Checkerboard assay

The antimicrobial activity of the molecules in combination a) with AMP against MRSA strains b) with NEO against NEO-resistant *E. coli* was tested using the method described by Zhu *et al*. ^39^ with some modifications. Briefly, overnight bacterial cultures were sub-cultured the following day and incubated until they reached the mid-exponential phase, corresponding to OD _600_ of 0.5–0.6. The bacterial suspension was then diluted to OD _600_ of 0.1 (equivalent to McFarland 0.5). Following this, 50 µL of the bacterial suspension was added to each well of a 96-well plate, containing 50 µL of MHB and the diluted molecules in combination with AMP or NEO in increasing concentrations. The fractional inhibitory concentration index (FICI) was calculated using the formula: FICI=FICA+FICB, where FIC _A_= MIC _A_ in combination/MIC _A_ alone and FIC _B_= MIC _B_ in combination/MIC _B_ alone. The FICI was used to categorize the interaction as synergistic (FICI ≤ 0.5), additive (FICI > 0.5 and ≤ 1.0), indifferent (FICI > 1.0 and ≤ 4.0) or as antagonistic (FICI > 4.0).

### Data analysis and growth inhibition calculation

To calculate the growth percentage of the bacteria at a certain condition, the OD _600_ values obtained from the plate reader were first blank-corrected using the average blank value of the MHB media (without bacteria/compounds), which was recorded in column 12 of the 96-well plate (plate set-up in Figure S1). After blank correction, the average full growth (with and without 1/8 MIC of the corresponding aminoglycoside) of *E. coli* and MRSA in the absence of validation compounds or bacterial extracts was calculated by averaging the values from column 11. This value represented 100% growth for each pathogen under specific conditions. The percentage of growth for each well was then calculated by dividing the OD _600_ of each well by the average full growth and multiplying by 100 to determine the growth percentage in the presence of the different antimicrobial compounds or extracts. For *E. coli*, the percentage of growth was calculated with and without 1/8 MIC of the corresponding aminoglycoside. To assess the helper-compound activity of each extract, the difference between the growth percentages with and without 1/8 MIC of the aminoglycoside was calculated. A higher positive difference indicated more pronounced helper-drug activity. For downstream hit selection, a threshold of ≥80% inhibition or enhancement was used to define positive candidates. This empirical cut-off was based on preliminary experiments to balance sensitivity and specificity in detecting meaningful effects. Chemical structures were drawn using ChemDraw Cloud (PerkinElmer Informatics, Waltham, MA, USA; https://chemdraw.perkinelmer.cloud).

## Results

### Growth of aminoglycoside-resistant *E. coli* in presence of the corresponding aminoglycoside

The MICs of NEO, STREP and GEN against aminoglycoside-resistant *E. coli* MG1655 strains were 2000, 1400 and 100 mg/L, respectively. To determine a baseline for further experiment, the average growth of the aminoglycoside-resistant *E. coli* without and with 1/8 MIC of the corresponding antibiotic was determined ( Table 1). 1/8 MIC of the corresponding aminoglycoside inhibited the growth of each aminoglycoside-resistant strains slightly, resulting in a growth of 72 – 90% compared to the growth without aminoglycosides.

**Table 1.** Growth of aminoglycoside-resistant strains with and without 1/8 MIC of the corresponding antibiotic.

	Aminoglycoside concentration (in mg/L)	OD _600_ control	OD _600_ with 1/8 MIC aminoglycoside	Growth with aminoglycosides in % (vs. control)
MG1655 *aph(3’)-Ia*	NEO: 250	1,16 (+/- 0,048)	0,84 (+/- 0,113)	72 % (+/- 8,2)
MG1655 *strAB*	STREP: 175	1,16 (+/- 0,032)	1,04 (+/- 0,081)	90 % (+/- 4,8)
MG1655 *aac(3)-IV*	GEN: 12,5	1,23 (+/- 0,027)	0,94 (+/- 0,096)	77 % (+/- 8,06)

### Assessing known antimicrobial compounds against *E. coli* and MRSA strains

**Figure 2.**
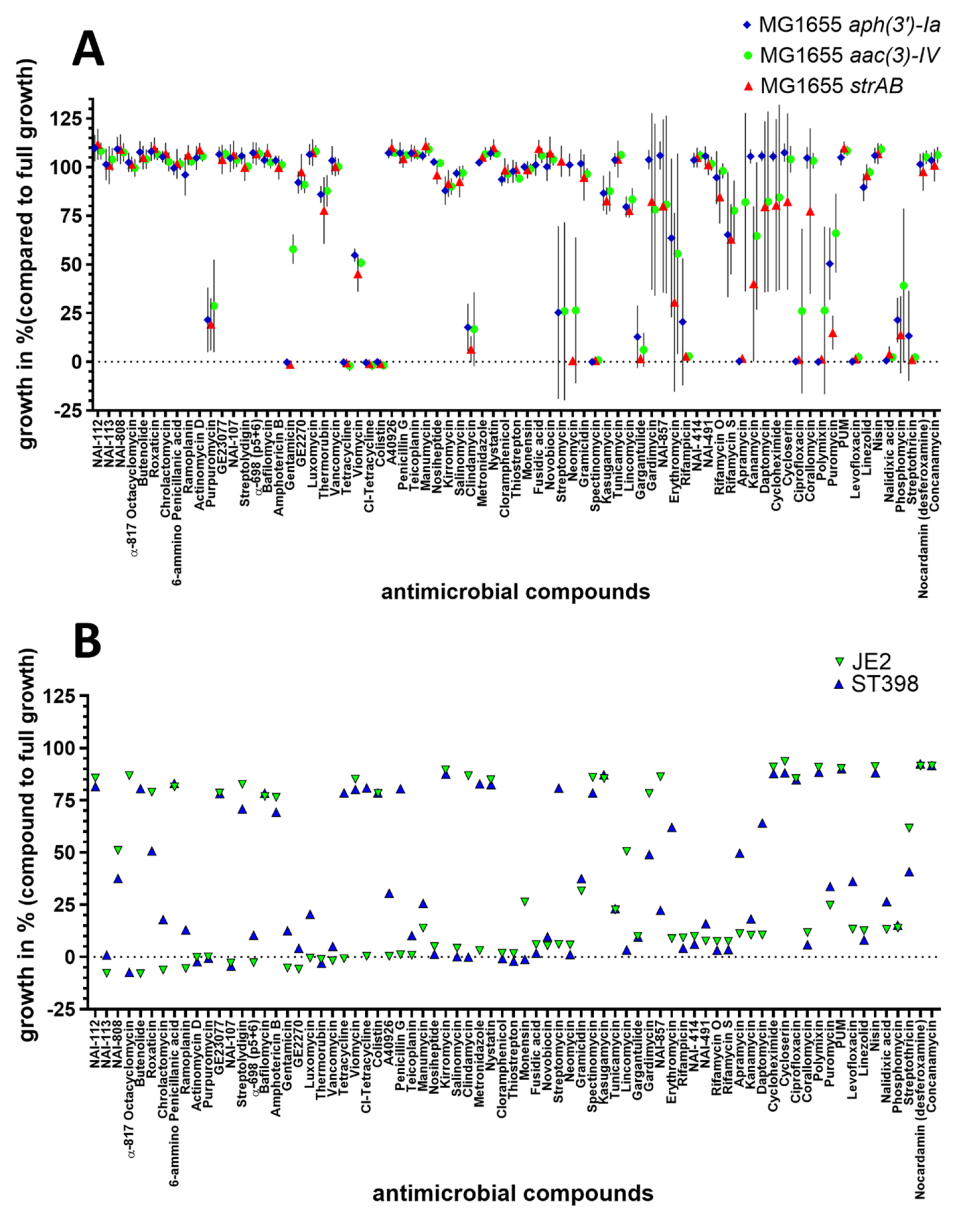
Growth inhibition of 75 compounds with antimicrobial activity on aminoglycoside-resistant *E. coli* and MRSA. NEO-resistant *E. coli*, STREP-resistant *E. coli*, and GEN-resistant *E. coli* ( **A**), MRSA ST398 and JE2 ( **B**). The bacterial strains were exposed to 75 different antimicrobial compounds. The antimicrobial compounds were present in a 96-well plate (Table S1 and S4) The average percentage of growth in the presence of the antimicrobial (compared to average growth without antimicrobials) was assessed in four biological replicates. The average growth and standard deviation were calculated using Excel and depicted with GraphPad Prism 10 (GraphPad Software, San Diego, USA).

To assess antimicrobial activity, we screened a library of 75 compounds with known antimicrobial properties against *E. coli* and MRSA strains (Table S1, S4 and Figure 2). Compounds that inhibited the growth of at least two aminoglycoside-resistant *E. coli* by more than 90 % included tetracyclines, polymyxins, the aminoglycosides GEN, spectinomycin and apramycin, fluoroquinolones, streptothricin and gargantulide ( Figure 2A). Furthermore, purpuromycin, clindamycin, phosphomycin and the aminoglycosides NEO and STREP partly inhibited the growth (>50% inhibition) of at least two of the aminoglycoside-resistant *E. coli* ( Figure 2A). The full list of compounds and the corresponding percentage of growth is listed in Table S1.

When screened against MRSA, most compounds demonstrated similar inhibition profiles for both ST398 and JE2 strains, though some notable differences were observed. As shown in Figure 2B, 25 out of the 75 compounds achieved at least 90% growth inhibition in ST398, while 34 compounds had the same level of inhibition against JE2. However, 12 compounds exhibited significant differential activity (≥50% difference) between the two strains.

The polyethers α-817 octacyclomycin, the lincosamides clindamycin and lincomycin, and NAI-857 were all more effective against ST398 than against JE2. Similarly, tetracycline and chlorotetracycline, butanolide, the b-lactam penicillin G, metronidazole, the aminoglycoside STREP, the macrolide erythromycin, and daptomycin all exhibited stronger activity against JE2 than against ST398. Of note, these effects were not class-specific: for example, streptomycin exhibited much stronger activity against JE2 (~6% growth) compared to ST398 (~80.8% growth), while neomycin showed minimal difference, with around 1.1% growth for ST398 and 5.77% for JE2. Full profiles of each compound against both strains (growth percentage) are presented in Table S4.

### Assessing helper-compound activity of compounds with antimicrobial activity for treatment of aminoglycoside-resistant *E. coli* MG1655

**Figure 3.**
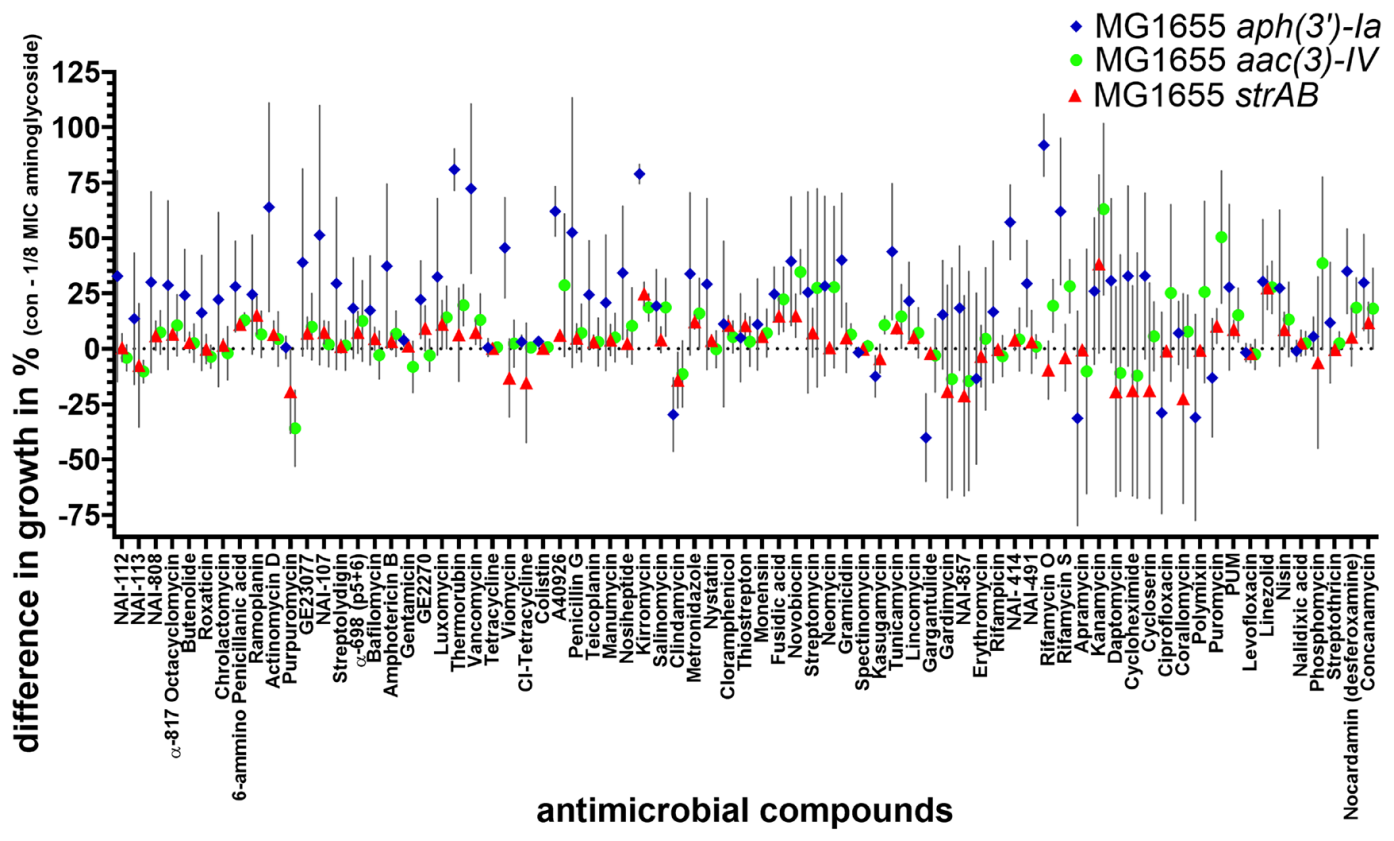
Testing of 75 compounds with known antimicrobial activity for potential aminoglycoside helper-compound activity NEO-resistant ( **blue**), GEN-resistant ( **green**) STREP-resistant ( **red)** MG1655 strains were exposed to 75 different antimicrobial compounds (final concentration: 20 mg/L). The antimicrobial compounds were present in a 96-well plate (Table S2). The difference in the percentage of growth with and without the aminoglycoside at 1/8 MIC was calculated by Excel (average of 4 biological replicates). The average and standard deviation were depicted using GraphPad Prism 10 (GraphPad Software, San Diego, USA).

After investigating which compounds inhibited the growth of aminoglycoside-resistant *E. coli* on their own, we tested which antimicrobials could act as aminoglycoside helper-compounds, i.e., they inhibited the growth of resistant *E. coli* in the presence of 1/8 MIC of the corresponding aminoglycoside. The difference in growth between the control (with 20 mg/L antimicrobial compounds) and treatment (with 20 mg/L antimicrobial compound and 1/8 MIC of the corresponding aminoglycoside) is presented in Figure 3. A good helper-compound would show a big difference between the two groups, since the inhibition by the compound alone would be small while the inhibition by the compound in combination with 1/8 MIC of the corresponding aminoglycoside would be considerable. This identified two antibiotics that caused an average difference in growth of more than 80% in four biological replicates in the presence of 1/8 MIC of NEO ( Figure 3). On their own, rifamycin O (20 mg/L) and thermorubin (20 mg/L) have no antimicrobial activity against NEO-resistant *E. coli* with an average OD _600 _of around 1 similar to full growth without the corresponding antimicobials. Combined with 1/8 MIC of NEO, the growth was considerably reduced with an OD _600_ of 0,02 for rifamycin O and 0,045 for thermorubin ( Figure 3). The synergist interaction between NEO and thermorubin (FICI: 0.25-0.375) as well as NEO and rifamycin O (FICI: 0.188-0.375) was confirmed by checkerboard assay. In combination with 1/8 MIC of NEO, 4–8 mg/L thermorubin (the MIC of thermorubin alone was 32–64 mg/L) and 16–32 mg/L rifamycin O (the MIC of rifamycin O alone was 128 mg/L) inhibited the growth of the NEO-resistant *E. coli*. Hence, the addition of 1/8 MIC of NEO led to an 8-fold reduction in MIC of thermorubin and 4-8-fold reduction in MIC of rifamycin O.

For GEN and STREP, no promising helper-compounds were identified; the helper-drug candidate for GEN and STREP with the highest synergistic activity was the aminoglycoside antibiotic kanamycin ( Figure 3) with an average difference of 63% and 38%, compared to growth in the presence of kanamycin alone.

### Assessing antimicrobial and helper-compound activity of *Actinomycetes* extracts

**Figure 4.**
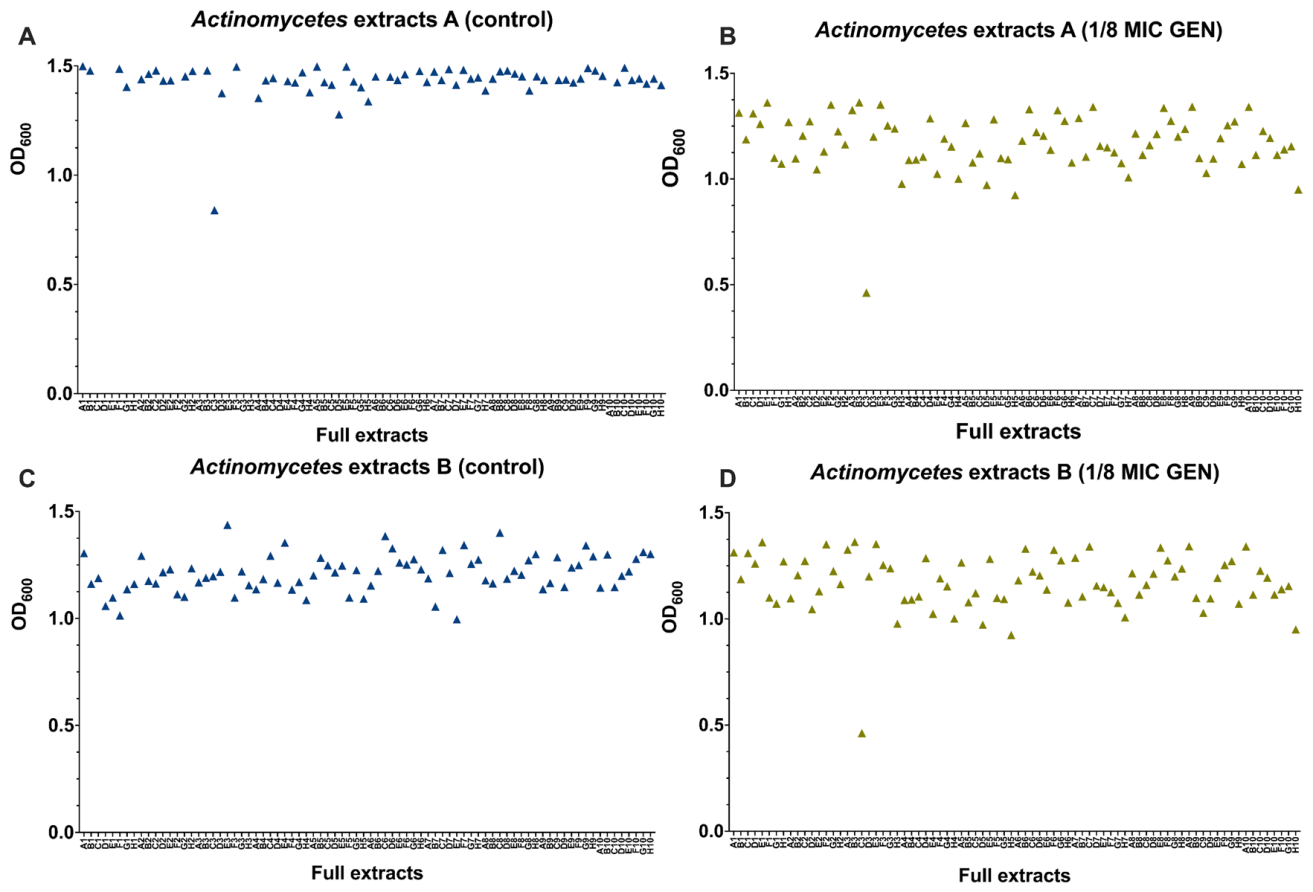
Screening of *Actinomycetes* extracts for antibacterial and helper-compound activity. 880 *Actinomycetes* extracts (shown are 160 extracts) were tested and for their antimicrobial activity ( **A**, **C**) and as helper-compounds in combination with 1/8 MIC of GEN ( **B**, **D**) against GEN-resistant *E. coli* by measuring their OD _600_ and depicted with GraphPad Prism 10 (GraphPad Software, San Diego, USA). The *Actinomycetes* extracts were present in a 96-well plate and the letter (A–H) combined with the number (1–10) referred to the position of each extract.

We screened 880 *Actinomycetes* extracts from the NAICONS library as described previously by Simone *et al*. ^40^. To identify those with antimicrobial or helper-compound activity against MRSA and GEN-resistant *E. coli*. Given GEN’s critical role in human health, we prioritized its use for helper-compound analysis. No extract inhibited the growth on their own nor in the presence of 1/8 MIC of GEN by more than 50 % ( Figure 4). However, one extract with moderate antimicrobial and one with moderate helper-compound activity were identified. One extract from a *Streptomyces* strain ^41^, showed a partial growth inhibition with and without 1/8 MIC of GEN ( **C3 in** Figure 4 A, B), indicating that this extract contains antimicrobial compound(s). Mass spectrometry analysis of this extract revealed that molecules belonging to the class of streptothricin were present in this extract ^41^ and streptothricin was shown to inhibit growth of aminoglycoside-resistant *E. coli* in the screen of compounds with known antimicrobial activity reported above ( Figure 4, **H3**). In addition, one extract from *Actinomadura* ^41^ showed no general antibacterial activity but helper-drug activity in combination with 1/8 MIC of GEN against the GEN-resistant *E. coli* ( **C3 in** Figure 4, C, D). A follow-up investigation revealed that this extract showed moderate concentration-dependent GEN helper-compound activity, since this extract on its own did not inhibit growth and the largest effect in growth inhibition was observed with 20 μL of extract (Figure S2). Mass spectrometry analysis for this extract ID 57183 did not show the presence of any known molecule, presenting the need for further experiments.

**Figure 5.**
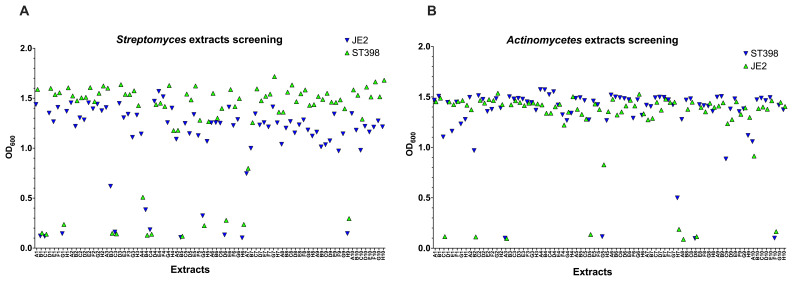
Screening of *Actinomycetes* extracts for antibacterial activity. 160 *Actinomycetes* extracts (80 from *Actinomycetes* rare extracts and 80 from *Streptomyces* strain) were tested for their antimicrobial activity. 10 hits were found in common from *Streptomyces* extracts ( **A**) while 4 hits were found in common from *Actinomyces* rare extracts ( **B**).

Additionally, we tested 80 *Streptomyces* extracts and 80 extracts from other *Actinomycetes* genera against ST398 and JE2 MRSA strains ( Figures 5A, and 5B respectively). Among the *Streptomyces* extracts, we found 10 hits that inhibited the growth of both strains by 90%. Further analysis via serial dilution confirmed the antimicrobial efficacy of two extracts, ( **D6)** and ( **H6)**, which demonstrated consistent 100% inhibition in at least one or two dilutions. From the other *Actinomycetes* extracts, we found four hits that inhibited both strains equally, with one ( **D8**) displaying potent antimicrobial activity over three successive dilutions. Based on these findings, extracts **D6** (Extract ID 44876), **H6** (Extract ID 38424), and **D8** (Extract ID 59998) were prioritized for further investigation.

**Figure 6.**
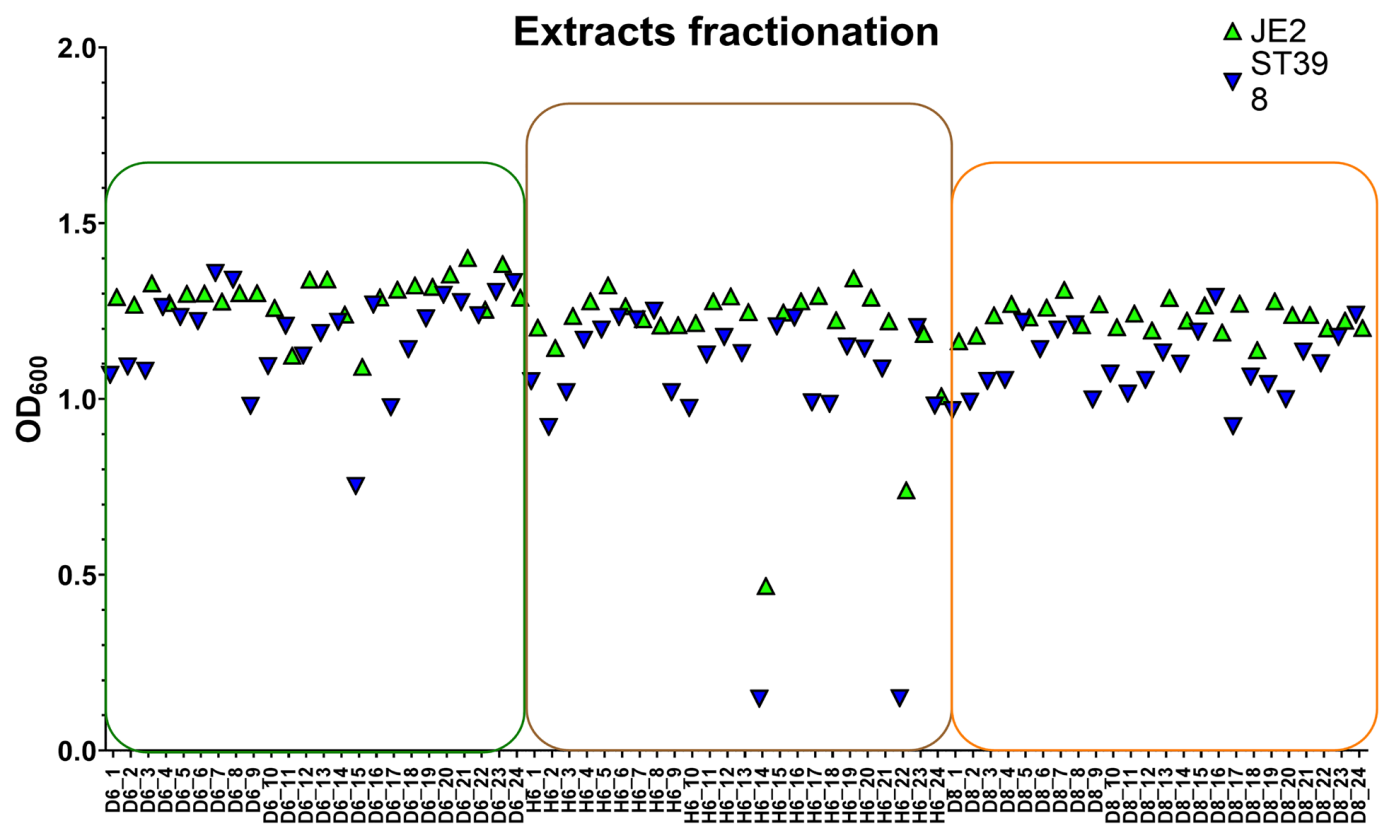
Scatter plots showing the measurement of OD _600_ of strains ST398 and JE2 exposed to 3 x 24 subfractions of each extract ( **D6**), ( **H6**), and ( **D8**) obtained through fractionation in the HPLC system (each extract was fractioned into 24 fractions). D6 subfractions are shown in green box, H6 in brown, and D8 in orange.

To understand better the activity profile of each extract, we generated six fractionated extracts (three from mycelium and three from cleared broth). The ( **D6**) extract showed notable activity in fraction 4 of the cleared broth across four serial dilutions, while ( **H6**) exhibited activity in fraction 5 of the cleared broth against both strains. Likewise, fraction 5 of the ( **D8**) extract displayed strong antimicrobial effects. These active fractions were subsequently analyzed by HPLC, generating sub-fractions for detailed testing against MRSA strains. The results of screening the 72 sub-fractions are shown in Figure 6 for both strains. Among the sub-fractions, **D6** (sub-fraction containing A1) inhibited ST398 by 40% and JE2 by 10.4%. **H6** fractions 14 and 22 showed high activity, inhibiting ST398 by 96% and JE2 by 65% and 41%, respectively. In contrast, sub-fractions of **D8** showed no significant activity.

### Identification of compounds by mass spectrometry analysis

After detecting the active fractions with antimicrobial activity, we proceeded with characterization using mass spectrometry. The first fraction analyzed, designated as A1, exhibited signals at **446.3112** in positive ion mode and **444.2968** in negative ion mode in the full scan. Upon fragmentation, two primary fragment ions were detected at **m/z 272.1855** and **m/z 106.0499**. These MS signals did not correspond to any compounds in the ChemNet or MassBANK databases, which contain extensive collections of known chemical structures and mass spectral data. The second fraction, labeled B1, exhibited signals at **m/z 742.5098** and **m/z 747.4648** in the full scan, corresponding to the [M+NH4]+ and [M+Na]+ adducts, respectively. MS/MS analysis revealed fragmentation patterns consistent with the molecule nigericin, as previously described by Harvey *et al*. ^42^. The fragmentation data are shown in Figure S3A, and the 2D structure of nigericin is provided in Figure S3B.

For the third fraction, B2, a precursor ion was detected at **m/z 1047.5850** in the full scan. MS/MS analysis revealed fragment ions at **m/z 729.3819**, **m/z 711.3713**, and **m/z 734.3972**, which are characteristic of elaiophylin, as previously reported by Boya *et al*. ^43^. The fragmentation spectra are displayed in Figure S4A, and the 2D structure of elaiophylin is shown in Figure S4B.

After confirming the activity of the molecules, by literature search, we found that both elaiophylin and nigericin are hexaene antibiotics produced by the microorganism *Streptomyces hygroscopicus* CH-7 ^44^ with reported activity against MRSA (Gui *et al*., 2019; Zhu *et al*., 2022). Importantly, the identification of nigericin and elaiophylin was based not only on accurate mass measurements but also on matching the fragmentation patterns (daughter ions) with those reported in the literature, thereby strengthening the confidence in our compound assignments. Both compounds are well-characterized hexaene antibiotics produced by Streptomyces hygroscopicus CH-7 (Ilic *et al.*, 2016) and have documented antimicrobial activity against MRSA strains (Gui *et al.*, 2019; Zhu *et al.*, 2022).

### Antimicrobial susceptibility testing

The MIC of elaiophylin was 0.75 µg/mL against ST398 and 1 µg/mL against JE2. For nigericin, the MIC values were slightly lower at 0.5 µg/mL and 0.75 µg/mL, against ST398 and JE2, respectively. In contrast, AMP (B) exhibited much higher MICs of 12 µg/mL for ST398 and 6 µg/mL for JE2.

When elaiophylin was tested in combination with AMP, the FICI obtained was 2.22 for ST398 and 2.46 for JE2, indicating no synergistic effect between the two antimicrobials. Similarly, combining nigericin with AMP resulted in a FICI value of 2.33 for ST398 and 3.33 for JE2, showing, even in this case, no synergistic interaction between these compounds as shown in Table 2.

**Table 2.** The MICs of elaiophylin and nigericin alone and in the presence of AMP.

Strain	Molecule A	MIC A (µg/mL)	MIC B (µg/mL)	FIC _A_	FIC _B_	FICI	*Effect*
*ST398*	Elaiophylin	0.75	12.00	1.22	1.00	2.22	Indifferent
	Nigericin	0.5	12.00	1.33	1.00	2.33	Indifferent
*JE2*	Elaiophylin	1.00	6.00	1.13	1.33	2.46	Indifferent
	Nigericin	0.75	6.00	2.00	1.33	3.33	Indifferent

MIC A: Minimum inhibition concentration of the tested molecule (elaiophylin/nigericin)

MIC B: Minimum inhibition concentration of AMP

FICI: fractional inhibitory concentration index of molecule A together with molecule B

## Discussion

### Helper drug screening against *E. coli*

In this study, we have established an *in vitro* screening approach that can be utilized for antimicrobial as well as antibiotic helper-compound identification. At first, we used a library of 75 known antibiotics against *S. aureus* and aminoglycoside-resistant *E. coli*. The classes of antibiotics that showed antimicrobial activity against *E. coli* include tetracyclines, aminoglycosides, fluoroquinolones, polymyxins and streptothricin, all already known for their activity against Gram-negative bacteria ^45– 47^. In addition to compounds with antibiotic activity, we set out to identify aminoglycoside helper-compounds. We selected the class of aminoglycosides since these antimicrobials are highly potent against Gram-negative bacteria, and they show good penetration through the Gram-negative double membrane structure ^13, 46^. However, due to an increasing level of resistance and toxicity concerns, aminoglycosides are less frequently used nowadays ^12, 13^. The application of helper-drugs could address both problems: a helper-drug administered in combination with aminoglycosides could re-sensitize aminoglycoside-resistant bacteria to treatment, and the combination would allow a lower concentration of aminoglycosides decreasing the risk of toxic side effects ^48, 49^. We performed the helper-drug screen at a concentration of 1/8 MIC of the corresponding aminoglycoside antibiotic as this concentration showed a growth inhibition of 10–28%. A lower concentration of the aminoglycoside would decrease the likelihood of finding a helper-compound and a higher concentration might be toxic enough to preclude further developments.

The aminoglycoside helper-drug screen with known antimicrobials, identified two antibiotics acting synergistically with NEO, which was confirmed by checkerboard assay. Among these, rifamycin inhibits the bacterial DNA-dependent RNA polymerase, showing limited activity against Gram-negatives ^50, 51^. Thermorubin interferes with protein biosynthesis by binding to the ribosomal 70S initiation complex and possesses activity against Gram-positives and selected Gram-negative bacteria including *E. coli* ^52, 53^. Rifamycin O and thermorubin did not show antimicrobial activity against *E. coli*, at the concentration used (20 mg/L), which is in line with previous studies where the MIC of rifamycin O was 75 mg/L ^50^. However, for thermorubin activity against *E. coli* SKF 12140 has been reported at this concentration ^53^. This discrepancy could be due to strain differences, and further studies are needed given the fact that resistance to thermorubin has not been well studied ^52, 53^. To the best of our knowledge, a synergistic combination of aminoglycosides and rifamycin O or thermorubin has not been reported previously. The reason for the observed synergy is currently unknown. A possible explanation is that rifamycin O and thermorubin increase the uptake of NEO or vice versa, requiring further investigations.

In addition to the screen with known antibiotics, we investigated the antimicrobial as well as GEN helper-compound activity of extracts from *Actinomycetes.* The choice of *Actinomycetes* extracts was based on their record as valuable resources in natural product discovery ^26, 47, 54– 56^. However, so far *Actinomycetes* extracts, including the extracts prepared by Naicons ^41^, have mostly been screened for direct antimicrobial activity ^56^, leaving room for discoveries of molecules with helper-compound activity ^23, 57, 58^.

One *Streptomyces* extract with antimicrobial activity was identified (extract ID 36463), most likely resulting from the presence of streptothricin, which has been reported to be the most common class of molecules responsible for antimicobial activity against Gram-negative bacteria identified in previous NAICONS screens ^56^. Additionally, one extract from *Actinomadura* (extract ID 57183) showed a moderate, concentration-dependent helper-compound activity, however, the responsible molecule needs to be identified. Nevertheless, the identification of helper-drugs and helper-extracts, despite the limited number of tested samples, confirms the applicability of our helper-drug screening approach.

### Compound and extract screening against *S. aureus*

Screening of 75 compounds against *S. aureus* revealed that the strains we tested were susceptible to paramagnetoquinones. The compound showed great efficacy with a 95% growth inhibition against JE2 and 88% against ST398. This compound, isolated from *Actinoallomurus*, has previously shown activity against with a low MIC of 0.015 μg/mL against Gram-positive pathogens ^59^. Here we confirm the activity of the molecule against MRSA strains relevant for humans and livestock. Another highly active compound was NAI-107, which inhibited ST398 and JE2 with a 94% inhibition rate. NAI-107, a lantibiotic known for its effectiveness against Gram-positive bacteria, including vancomycin-resistant *S. aureus*, disrupts cell wall formation, leading to the accumulation of the soluble peptidoglycan precursor UDP-N-acetylmuramic acid-pentapeptide (UDP-MurNAc-pentapeptide) within the cytoplasm ^60^. Two additional compounds, enduracyclinones and NAI-414, demonstrated notable activity. Enduracyclinones exhibited 73 and 85% growth inhibition of ST398 and JE2, respectively. These compounds are supposed to inhibit both DNA and peptidoglycan biosynthesis, providing a dual mechanism of action against Gram-positive pathogens ^61^. NAI-414, with an 83% inhibition rate, belongs to the spirotetronate class and has demonstrated both anti-staphylococcal and anti-tumor activities ^62^. Generally, there were good similarities in the screening of anti-antimicrobial compounds between the two MRSA strains. However, we observed different activity by some compounds, with some being ineffective against one of them. This highlights the importance of strain selection before conducting screening.

Furthermore, two molecules, elaiophylin and nigericin, with activity against MRSA strains were identified. Elaiophylin, a macrodiolide, showed antimicrobial activity by inhibiting the NorA efflux pump in *S. aureus*, which appears to be overexpressed in *S. aureus* during biofilm growth ^63^. This mechanism causes a reduction and/or inhibition of bacterial growth, as well as creating pores in the lipid bilayer of bacterial membranes ^64^. Elaiophylin also has anti-cancer properties, including autophagy inhibition and antiangiogenic effects ^63^. Numerous studies have also confirmed that it could be used in combination with other molecules, such as norfloxacin and ethidium bromide, to enhance the effect of pharmacological treatments ^65^. The second molecule, nigericin, which reduces ATP production by interfering with the electron transport chain, is also known to have activity against Gram-positive bacteria ^39^. The pure molecules were tested alone and for synergistic effects with the β-lactam antibiotic AMP, to determine if they could be used as helper molecules in combination therapies. AMP is known to bind to penicillin-binding proteins, which play a role in the final steps of peptidoglycan synthesis in the bacterial cell wall ^66^. We hypothesized that disrupting the cell wall would enhance the penetration of these molecules, potentially reducing MIC values. However, no synergistic effect was observed, indicating that elaiophylin and nigericin do not have an additive effect with AMP. Although elaiophylin does not show an additive effect with β-lactams, it has been demonstrated to exhibit a synergistic effect with fluoroquinolones, such as norfloxacin, which inhibit the DNA gyrase enzyme and impede DNA replication ^65, 67^.

In conclusion, despite the limited scope of our screening, which included 75 purified compounds and 160 (for MRSA)/880 (for *E. coli*) *Actinomycetes* extracts, we were able to identify several promising compounds with potential antimicrobial and antibiotic-helper activity that warrant further investigation. These findings highlight the importance and value of screening natural products for drug-like molecules. By utilizing a diverse library of natural compounds, we demonstrate the capacity to discover novel synergistic interactions and helper-drug candidates, further supporting the potential of *Actinomycetes* as a rich resource for future therapeutic development. The results from this initial screening highlight the advantage of expanding the search for new antibiotics and helper compounds beyond traditional synthetic libraries, which may lead to more effective combination therapies for treating resistant bacterial infections. However, the screening also confirms that more antibiotics are effective against Gram-positive bacteria than Gram-negative bacteria, as previously highlighted ^68^.

## Ethics and consent

Ethical approval and consent were not required.

## Data Availability

Repository name: Open Science Framework: Screening Actinomycetes extracts for antimicrobial compounds against methicillin-resistant *Staphylococcus aureus* and helper-compounds against aminoglycoside-resistant *E. coli.* https://doi.org/10.17605/OSF.IO/SQ6GZ ^69^. The project contains the following underlying data: Metadata_results.xlsx (Contains all the meta results provided in the manuscript). Repository name: Open Science Framework: Screening Actinomycetes extracts for antimicrobial compounds against methicillin-resistant *Staphylococcus aureus* and helper-compounds against aminoglycoside-resistant *E. coli.* https://doi.org/10.17605/OSF.IO/SQ6GZ ^69^. The project contains the following underlying data: Supplementary_file.pdf (Contains all tables and figures presented in the text as supplementary file). Data are available under the terms of the Creative Commons Attribution 4.0 International license (CC-BY 4.0).
