# Supplementary material for: Screening Actinomycetes extracts for antimicrobial compounds against methicillin-resistant Staphylococcus aureus and helper-compounds against aminoglycoside-resistant E. coli
Source: Open Res Eur. 2025 Aug 11;5:110. Originally published 2025 Apr 17. [Version 2] doi: 10.12688/openreseurope.19988.2 (PMC12417992; doi:10.12688/openreseurope.19988.2)
Supplement: Peer review history

# Reviewer report: Reviewer response for version 2

**DOI:** 10.21956/openreseurope.22663.r58980

**Published:** 2025-09-15

**Authors:** Galal Yahya

**Affiliations:** 1 Molecular Biology Institute of Barcelona (IBMB), Barcelona, Spain; 2 Microbiology and Immunology, Zagazig University Faculty of Pharmacy (Ringgold ID: 110192), Zagazig, Ash Sharqia Governorate, Egypt

I have the following concerns regarding the study

 1- The abstract should provide quantitative outcomes

 2- The rationale for selecting aminoglycosides (helper-drug focus) is present but should more clearly justify *clinical urgency*

 3- The novelty of combining known antibiotics with Actinomycete extracts is underplayed.

 4- Literature on *previous helper-compound discoveries* is only briefly referenced; should better position this work in the drug discovery landscape.

 5- Strain selection is justified (community vs. livestock MRSA), but no rationale for *only one aminoglycoside-resistant E. coli background strain* (MG1655) – may limit generalizability.

 6- Checkerboard assays confirm synergy, but the choice of 1/8 MIC concentration could be better defended.

 7- Screening thresholds (≥80% inhibition/enhancement) are empirically set, but justification remains somewhat weak

 8- Data presentation is sometimes too descriptive; trends are buried in text rather than emphasized with comparative analysis.

 9- Negative findings (e.g., lack of synergy with ampicillin) are reported but under-discussed.

 10- For MRSA, differences between JE2 and ST398 are noted, but mechanistic implications of strain-specific susceptibilities are not explored.

 11- Figures could be clearer: Some strain labels/colors (Figures 5A/5B) were corrected after review, but others remain hard to interpret.

 12- No mention of toxicity concerns for identified molecules (nigericin, elaiophylin are cytotoxic in mammalian systems).

 13- Mechanistic speculation on rifamycin/thermorubin synergy is very limited (“possibly uptake-related”) — this needs expansion.

 14- The role of strain-specific susceptibility differences is under-discussed.

Is the study design appropriate and does the work have academic merit?

Yes

Is the work clearly and accurately presented and does it cite the current literature?

Yes

If applicable, is the statistical analysis and its interpretation appropriate?

I cannot comment. A qualified statistician is required.

Are all the source data underlying the results available to ensure full reproducibility?

Yes

Are the conclusions drawn adequately supported by the results?

Partly

Are sufficient details of methods and analysis provided to allow replication by others?

Yes

Reviewer Expertise:

Infection biology, immunology, cancer, inflammation

I confirm that I have read this submission and believe that I have an appropriate level of expertise to confirm that it is of an acceptable scientific standard, however I have significant reservations, as outlined above.

# Reviewer report: Reviewer response for version 2

**DOI:** 10.21956/openreseurope.22663.r58057

**Published:** 2025-09-08

**Authors:** Jonathan Parra

**Affiliations:** 1 Universidad de Costa Rica, San José, Costa Rica

After reviewing the authors’ responses to the requested revisions, I find the changes satisfactory.

Is the study design appropriate and does the work have academic merit?

Yes

Is the work clearly and accurately presented and does it cite the current literature?

Yes

If applicable, is the statistical analysis and its interpretation appropriate?

Not applicable

Are all the source data underlying the results available to ensure full reproducibility?

Partly

Are the conclusions drawn adequately supported by the results?

Yes

Are sufficient details of methods and analysis provided to allow replication by others?

Partly

Reviewer Expertise:

Metabolomics

I confirm that I have read this submission and believe that I have an appropriate level of expertise to confirm that it is of an acceptable scientific standard.

# Reviewer report: Reviewer response for version 2

**DOI:** 10.21956/openreseurope.22663.r58056

**Published:** 2025-08-28

**Authors:** K S Sunish

**Affiliations:** 1 Maharaja's College, Ernakulam, India

I have now carefully reviewed the revised version and the authors' point-by-point responses to my previous comments. I am pleased to confirm that the authors have addressed all of my queries satisfactorily and have made the necessary clarifications. Their revisions have substantially strengthened the manuscript, and I believe it now meets the journal's standards for indexing.

 Consequently, I am happy to recommend that the manuscript be accepted for indexed in its current form.

Is the study design appropriate and does the work have academic merit?

Yes

Is the work clearly and accurately presented and does it cite the current literature?

Yes

If applicable, is the statistical analysis and its interpretation appropriate?

Yes

Are all the source data underlying the results available to ensure full reproducibility?

Partly

Are the conclusions drawn adequately supported by the results?

Yes

Are sufficient details of methods and analysis provided to allow replication by others?

Partly

Reviewer Expertise:

NA

I confirm that I have read this submission and believe that I have an appropriate level of expertise to confirm that it is of an acceptable scientific standard.

# Reviewer report: Reviewer response for version 1

**DOI:** 10.21956/openreseurope.21619.r53724

**Published:** 2025-06-02

**Authors:** K S Sunish

**Affiliations:** 1 Maharaja's College, Ernakulam, India

The manuscript titled “ *Screening Actinomycetes extracts for antimicrobial compounds against methicillin-resistant Staphylococcus aureus and helper-compounds against aminoglycoside-resistant E. coli*” emphasizes the identification of promising antimicrobial compounds and antibiotic-helpers and underscores the value of natural product screening, particularly Actinomycetes, as a viable source for novel drug discovery, reinforcing the study's contribution to the field. The work suggests a meaningful direction for future research by advocating for expanded screening beyond synthetic libraries, which aligns with current needs in antibiotic development. This study provides a solid foundation for further exploration of natural product-derived antibiotics and adjuvants.

**The following points may also be clarified and revised before the manuscript is accepted for Indexing.**

1. While the numbers (75 compounds, 160/880 extracts) provide context, it might help to briefly contextualize why this is considered a "limited scope" (e.g., compared to larger high-throughput screenings).
2. Clarify whether the 880 extracts were tested against both MRSA and *E. coli* (the conclusion paragrapgh mentions 160 for MRSA and 880 for *E. coli*, creating potential ambiguity).
3. Provide quantitative data (e.g., fold potentiation, MIC reductions in mg/L or μM) for the observed adjuvant effects to substantiate these findings.
4. Certain methodological details require further clarification for reproducibility and better understanding. Please elaborate on the following: a) In the **Methods section**, under "Preparation of antimicrobial compound and extract plates" b) In the **Results section**, regarding "Assessing antimicrobial and helper-compound activity of *Actinomycetes* extracts.
5. Please address the following typographical errors, formatting issues, and points of clarity for overall manuscript quality: a) In the **Results section**, the sentence "Difference ‘is’ susceptibility towards antimicrobials and extracts were observed between the two MRSA strains used for screening." appears to contain a typographical or grammatical error. Please revise for clarity (e.g., "Differences *in* susceptibility... *were* observed..." or "A difference *in* susceptibility... *was* observed..."). b) In the **Methods section**, the sentence "Compounds and their corresponding solvents appear in ‘Table S1 and S2’" is noted. Please ensure these tables are correctly referenced, complete, and appropriately formatted. Consider whether their placement (main text versus supplementary data) is optimal for readability and impact. c) In the **Results section**, please correct the apparent typo: "antimicobials" should likely be "antimicrobials" in the sentence: "...full growth without the corresponding ‘antimicobials’." d) Throughout the manuscript, particularly noted in the **Results section** (e.g., OD600 values of ‘0,02’ for rifamycin O and ‘0,045’ for thermorubin), please ensure the consistent use of a period as the decimal separator (e.g., 0.02, 0.045) instead of a comma, to adhere to standard scientific conventions.

**After the above minor revisions, the manuscript may be accepted for Indexing.**

Is the study design appropriate and does the work have academic merit?

Yes

Is the work clearly and accurately presented and does it cite the current literature?

Yes

If applicable, is the statistical analysis and its interpretation appropriate?

Yes

Are all the source data underlying the results available to ensure full reproducibility?

Partly

Are the conclusions drawn adequately supported by the results?

Yes

Are sufficient details of methods and analysis provided to allow replication by others?

Partly

Reviewer Expertise:

Biotechnology & Microbiology

I confirm that I have read this submission and believe that I have an appropriate level of expertise to confirm that it is of an acceptable scientific standard, however I have significant reservations, as outlined above.

# Response

**Published:** 2025-07-28

**Authors:** Dina Nahhas

**Affiliations:** The University of Edinburgh School of Biological Sciences, Edinburgh, Scotland, UK

1. **While the numbers (75 compounds, 160/880 extracts) provide context, it might help to briefly contextualize why this is considered a "limited scope" (e.g., compared to larger high-throughput screenings).**

Au: Thank you for the comment. We acknowledge that our screening library is smaller than conventional high-throughput campaigns. However, the scope of the current work was determined by the availability of the extracts and time constraints. Our objective was to prioritize a targeted screen focusing on clinically relevant resistant strains ( *E. coli* and MRSA), with the intention of continuing downstream characterization once active hits were identified.

1. **Clarify whether the 880 extracts were tested against both MRSA and** ***E. coli*** **(the conclusion paragrapgh mentions 160 for MRSA and 880 for** ***E. coli*** **, creating potential ambiguity).**

Au: We appreciate this observation and agree it required clarification. We have now explicitly stated in both the Methods and Results sections (page 5, lines 218-223) that all 880 extracts were screened against *E. coli* for helper-compound activity, while a subset of 160 extracts was tested against MRSA for direct antimicrobial effects. The broader screen for *E. coli *reflects the greater challenge in identifying active compounds against Gram-negative bacteria. In contrast, MRSA screening was halted at 160 extracts due to early hit identification, allowing us to focus on subsequent purification and hit characterization steps.

1. **Provide quantitative data (e.g., fold potentiation, MIC reductions in mg/L or μM) for the observed adjuvant effects to substantiate these findings.**

Au: We thank the reviewer for their comment. To better illustrate the observed synergy between NEO and thermorubin/rifamycin O (these were the two hits identified in the adjuvant screen for which the synergistic effect was quantified), we have added an additional sentence including the observed MIC reduction. “In combination with 1/8 MIC of NEO, 4-8 mg/L thermorubin (the MIC of thermorubin alone was 32-64 mg/L) and 16-32 mg/L rifamycin O (the MIC of rifamycin O alone was 128 mg/L) inhibited the growth of the NEO-resistant E. coli. Hence, the addition of 1/8 MIC of NEO led to an 8-fold reduction in MIC of thermorubin and 4-8-fold reduction in MIC of rifamycin O. “ (page 10, line 367-371).  

1. **Certain methodological details require further clarification for reproducibility and better understanding. Please elaborate on the following: a) In the** **Methods section, under "Preparation of antimicrobial compound and extract plates" b) In the** **Results section, regarding "Assessing antimicrobial and helper-compound activity of** ***Actinomycetes*** **extracts.**
2. Au: We appreciate this suggestion. We have now expanded the “Preparation of antimicrobial compound and extract plates” section to include detailed steps on compound dilution, solvent use, storage conditions, and layout on the 96-well plates (page 4, lines 118–146), and the results section on "Assessing antimicrobial and helper-compound activity of Actinomycetes extracts to include details on (page 10 , lines. 367 - 371).

 

1. **Please address the following typographical errors, formatting issues, and points of clarity for overall manuscript quality: a) In the** **Results section, the sentence "Difference ‘is’ susceptibility towards antimicrobials and extracts were observed between the two MRSA strains used for screening." appears to contain a typographical or grammatical error. Please revise for clarity (e.g., "Differences** ***in*** **susceptibility...** ***were*** **observed..." or "A difference** ***in*** **susceptibility...** ***was*** **observed...") (could not find this sentence). b) In the** **Methods section, the sentence "Compounds and their corresponding solvents appear in ‘Table S1 and S2’" is noted. Please ensure these tables are correctly referenced, complete, and appropriately formatted. Consider whether their placement (main text versus supplementary data) is optimal for readability and impact (I think they are well referenced). c) In the** **Results section, please correct the apparent typo: "antimicobials" should likely be "antimicrobials" in the sentence: "...full growth without the corresponding ‘antimicobials’ (done)." d) Throughout the manuscript, particularly noted in the** **Results section** **(e.g., OD600 values of ‘0,02’ for rifamycin O and ‘0,045’ for thermorubin), please ensure the consistent use of a period as the decimal separator (e.g., 0.02, 0.045) instead of a comma, to adhere to standard scientific conventions (done).**

# Response

**Published:** 2025-08-05

**Authors:** John Elmerdahl Olsen

**Affiliations:** Veterinary and Animal Sciences, University of Copenhagen, Frederiksberg, Denmark

Comments: The manuscript titled “Screening Actinomycetes extracts for antimicrobial compounds against methicillin-resistant Staphylococcus aureus and helper-compounds against aminoglycoside-resistant E. coli” emphasizes the identification of promising antimicrobial compounds and antibiotic-helpers and underscores the value of natural product screening, particularly Actinomycetes, as a viable source for novel drug discovery, reinforcing the study's contribution to the field. The work suggests a meaningful direction for future research by advocating for expanded screening beyond synthetic libraries, which aligns with current needs in antibiotic development. This study provides a solid foundation for further exploration of natural product-derived antibiotics and adjuvants. The following points may also be clarified and revised before the manuscript is accepted for Indexing.

 Comment: While the numbers (75 compounds, 160/880 extracts) provide context, it might help to briefly contextualize why this is considered a "limited scope" (e.g., compared to larger high-throughput screenings).

**Authors response:** Thank you for the comment. We acknowledge that our screening library is smaller than conventional high-throughput campaigns. However, the scope of the current work was determined by the availability of the extracts and time constraints. Our objective was to prioritize a targeted screen focusing on clinically relevant resistant strains (E. coli and MRSA), with the intention of continuing downstream characterization once active hits were identified.

**Comment:** Clarify whether the 880 extracts were tested against both MRSA and E. coli (the conclusion paragrapgh mentions 160 for MRSA and 880 for E. coli, creating potential ambiguity).

**Authors response**: We appreciate this observation and agree it required clarification. We have now explicitly stated in both the Methods and Results sections (page 5, lines 218-223) that all 880 extracts were screened against E. coli for helper-compound activity, while a subset of 160 extracts was tested against MRSA for direct antimicrobial effects. The broader screen for E. coli reflects the greater challenge in identifying active compounds against Gram-negative bacteria. In contrast, MRSA screening was halted at 160 extracts due to early hit identification, allowing us to focus on subsequent purification and hit characterization steps. **Comment:** Provide quantitative data (e.g., fold potentiation, MIC reductions in mg/L or μM) for the observed adjuvant effects to substantiate these findings.

**Authors response**: We thank the reviewer for the comment. To better illustrate the observed synergy between NEO and thermorubin/rifamycin O (these were the two hits identified in the adjuvant screen for which the synergistic effect was quantified), we have added an additional sentence including the observed MIC reduction. “In combination with 1/8 MIC of NEO, 4-8 mg/L thermorubin (the MIC of thermorubin alone was 32-64 mg/L) and 16-32 mg/L rifamycin O (the MIC of rifamycin O alone was 128 mg/L) inhibited the growth of the NEO-resistant E. coli. Hence, the addition of 1/8 MIC of NEO led to an 8-fold reduction in MIC of thermorubin and 4-8-fold reduction in MIC of rifamycin O. “ (page 10, line 367-371).

**Comment:** Certain methodological details require further clarification for reproducibility and better understanding. Please elaborate on the following: a) In the Methods section, under "Preparation of antimicrobial compound and extract plates" b) In the Results section, regarding "Assessing antimicrobial and helper-compound activity of Actinomycetes extracts. **Authors response:** We appreciate this suggestion. We have now expanded the “Preparation of antimicrobial compound and extract plates” section to include detailed steps on compound dilution, solvent use, storage conditions, and layout on the 96-well plates (page 4, lines 118–146), and the results section on "Assessing antimicrobial and helper-compound activity of Actinomycetes extracts to include details on (page 10 , lines. 367 - 371). **Comment**: Please address the following typographical errors, formatting issues, and points of clarity for overall manuscript quality: In the Results section, the sentence "Difference ‘is’ susceptibility towards antimicrobials and extracts were observed between the two MRSA strains used for screening." appears to contain a typographical or grammatical error. Please revise for clarity (e.g., "Differences in susceptibility... were observed..." or "A difference in susceptibility... was observed...") **Authors response: **This sentence is no longer found in the manuscript **Comment: **Please address the following typographical errors, formatting issues, and points of clarity for overall manuscript quality: In the Methods section, the sentence "Compounds and their corresponding solvents appear in ‘Table S1 and S2’" is noted. Please ensure these tables are correctly referenced, complete, and appropriately formatted. Consider whether their placement (main text versus supplementary data) is optimal for readability and impact. **Authors response**: Thank you for the comment. We have considered this and we think they are well referenced. **Comment**: Please address the following typographical errors, formatting issues, and points of clarity for overall manuscript quality: In the Results section, please correct the apparent typo: "antimicobials" should likely be "antimicrobials" in the sentence: "...full growth without the corresponding ‘antimicobials’ (done)." d) Throughout the manuscript, particularly noted in the Results section (e.g., OD600 values of ‘0,02’ for rifamycin O and ‘0,045’ for thermorubin), please ensure the consistent use of a period as the decimal separator (e.g., 0.02, 0.045) instead of a comma, to adhere to standard scientific conventions. **Authors response**: Thank you. We have corrected this.

# Reviewer report: Reviewer response for version 1

**DOI:** 10.21956/openreseurope.21619.r53965

**Published:** 2025-06-02

**Authors:** Jonathan Parra

**Affiliations:** 1 Universidad de Costa Rica, San José, Costa Rica

The research article by Al Nahhas *et al*. describes the development of a screening platform for a collection of actinomycetes that aims to identify metabolites with antimicrobial and helper-compound activity. While natural products remain a particularly promising source for antibiotic discovery, screening large libraries of crude extracts remains a technological limitation. In this context, the development of a strategy for screening 880 Actinomycetes extracts and the subsequent identification of candidate metabolites responsible for biological activity is an important contribution that is worthy of being communicated.

 The use of 75 compounds with known antimicrobial activity for platform development is highly appropriate. However, there are certain aspects of the data presentation that would be worth improving for a better interpretation of the results. In particular, Figures 2 and 3 do not show error bars even though the experiments were performed with four biological replicates. For example, in the helper-compound activity test with compounds with known antibiotic activity, the authors report two hits (rifamycin O and thermorubin) with an average growth difference of over 80% in the neomycin assay. However, kirromycin showed an average difference in the growth of 78%, and it is not clear whether this 2% difference is significant or not. Additionally, the rationale for using 80% as a cut-off value was not completely explained.

 The methodological details of the LC-MS dereplication strategy are not entirely clear. It would be important to include information about the algorithm or criteria used to determine spectral matches. Figures S3A and S3B show the mass spectra; however, showing library reference spectra or, preferably, mirror plots would more effectively illustrate the spectral matches.

 In the discussion section, the reference to "purified molecules" when describing the activity of elaiophylin and nigericin is inaccurate, as the compounds were synthesised rather than purified from crude extracts. Therefore, it would be more accurate to refer to them as "pure compounds".

 Finally, the limitations of the screening strategy need to be discussed in more depth. In particular, the use of mass spectrometry for structural characterisation. The identified compounds (elaiophylin and nigericin) contain a large number of chiral centres; however, mass spectrometry is stereochemically blind, which raises some doubts about the true identity of the metabolites present in the crude extracts.

 Minor points:

 -Using the plate position instead of the Extract ID in the data plotting can be confusing for the reader.

 -Interchanging the strain colour in panels A and B of Figure 5 makes interpretation of the results difficult.

 -Depositing the mass spectrometry data in a repository like MassIVE would greatly benefit open science. However, the context of a private collection is understandable.

Is the study design appropriate and does the work have academic merit?

Yes

Is the work clearly and accurately presented and does it cite the current literature?

Yes

If applicable, is the statistical analysis and its interpretation appropriate?

Not applicable

Are all the source data underlying the results available to ensure full reproducibility?

Partly

Are the conclusions drawn adequately supported by the results?

Yes

Are sufficient details of methods and analysis provided to allow replication by others?

Partly

Reviewer Expertise:

Metabolomics

I confirm that I have read this submission and believe that I have an appropriate level of expertise to confirm that it is of an acceptable scientific standard, however I have significant reservations, as outlined above.

# Response

**Published:** 2025-07-28

**Authors:** Dina Nahhas

**Affiliations:** The University of Edinburgh School of Biological Sciences, Edinburgh, Scotland, UK

**Au:** Thank you for your comment. We have revised Figures 2 and 3 to include error bars representing standard deviation across four biological replicates, to better reflect the variability of the data.   This threshold was empirically defined during pilot screening experiments as a balance point between sensitivity and specificity—minimizing false positives while retaining potential hits for further validation. While kirromycin showed an average of 78% inhibition, the variability between replicates placed it below our cut-off when considering standard deviation. We have now included a detailed explanation of this rationale in the revised manuscript (Page 7, Line 299-302). 

**Au**: Extract 44916 and its fractions were submitted to the automatic annotation workflow of the Molecules Gateway*, based on proprietary developed software that combines and ranks the annotation results from Compound Discoverer, MS2Query and MolDiscovery. The proposed annotation for Nigericin, annotated by MS2Query with score 0.95, was manually confirmed by comparison with a commercial standand (ADIPOGEN AG-CN2-0020) and mirror plot is provided in **figure S3A**. Elaiophilin, for which none of the software was able to provide annotation, was manually annotated by comparison with published data** and experimental MS/MS from GNPS public database. Mirror plot is provided in **figure S4A**. *Simone M, Iorio M, Monciardini P, Santini M, Cantù N, Tocchetti A, Serina S, Brunati C, Vernay T, Gentile A, Aracne M, Cozzi M, van der Hooft J, Sosio M, Donadio S, Maffioli SI. “The molecules Gateway: A Homogeneous, Searchable Database of 150k Annotated Molecules from Actinomycetes” Journal of Natural Products, 2024,  87, 11, 2615–2628, https://doi.org/10.1021/acs.jnatprod.4c00857 **(biblio ref already present)    

**Au: **We thank the reviewer for highlighting the stereochemical limitations of mass spectrometry. Indeed, MS/MS alone does not provide direct stereochemical information. However, in our study, metabolite identification was supported by multiple complementary criteria: high-resolution accurate mass measurements, detailed fragmentation patterns, retention time alignment, and crucially, the matching of daughter ion spectra with those reported in the literature for known compounds like elaiophylin and nigericin. These combined pieces of evidence offer a robust and reliable annotation of the metabolites in our extracts. While techniques such as NMR or chiral chromatography are necessary for full stereochemical resolution and discovery of novel stereoisomers, the MS-based dereplication approach used here is well accepted for identifying known natural products and suffices to support the conclusions of our screening platform. We clarified this point in the manuscript to accurately convey both the strengths and limitations of our identification strategy (page 14 line 467-472).

**Minor points:**

**-Using the plate position instead of the Extract ID in the data plotting can be confusing for the reader. ** Thank you for the suggestion. However, we think that the name of the extract would be too long to read on the plot, in addition, they are provided in the supplementary table.

**-Interchanging the strain colour in panels A and B of Figure 5 makes interpretation of the results difficult. **(now has been changed)

# Response

**Published:** 2025-08-05

**Authors:** John Elmerdahl Olsen

**Affiliations:** Veterinary and Animal Sciences, University of Copenhagen, Frederiksberg, Denmark

**Comment:** The research article by Al Nahhas et al. describes the development of a screening platform for a collection of actinomycetes that aims to identify metabolites with antimicrobial and helper-compound activity. While natural products remain a particularly promising source for antibiotic discovery, screening large libraries of crude extracts remains a technological limitation. In this context, the development of a strategy for screening 880 Actinomycetes extracts and the subsequent identification of candidate metabolites responsible for biological activity is an important contribution that is worthy of being communicated. The use of 75 compounds with known antimicrobial activity for platform development is highly appropriate. However, there are certain aspects of the data presentation that would be worth improving for a better interpretation of the results. In particular, Figures 2 and 3 do not show error bars even though the experiments were performed with four biological replicates. For example, in the helper-compound activity test with compounds with known antibiotic activity, the authors report two hits (rifamycin O and thermorubin) with an average growth difference of over 80% in the neomycin assay. However, kirromycin showed an average difference in the growth of 78%, and it is not clear whether this 2% difference is significant or not. Additionally, the rationale for using 80% as a cut-off value was not completely explained.

**Authors response**: Thank you for the comment. We have revised Figures 2 and 3 to include error bars representing standard deviation across four biological replicates, to better reflect the variability of the data. Variation with screening for activity against Staphylococcus was minimal, and the bars depicting variation can hardly been seen in the figure, though. The threshold for important activity was empirically defined during pilot screening experiments as a balance point between sensitivity and specificity—minimizing false positives while retaining potential hits for further validation. While kirromycin showed an average of 78% inhibition, the variability between replicates placed it below our cut-off when considering standard deviation. We have now included a detailed explanation of this rationale in the revised manuscript (Page 7, Line 299-302).  

**Comment**: The methodological details of the LC-MS dereplication strategy are not entirely clear. It would be important to include information about the algorithm or criteria used to determine spectral matches. Figures S3A and S3B show the mass spectra; however, showing library reference spectra or, preferably, mirror plots would more effectively illustrate the spectral matches. Authors response: Extract 44916 and its fractions were submitted to the automatic annotation workflow of the Molecules Gateway*, based on proprietary developed software that combines and ranks the annotation results from Compound Discoverer, MS2Query and MolDiscovery. The proposed annotation for Nigericin, annotated by MS2Query with score 0.95, was manually confirmed by comparison with a commercial standand (ADIPOGEN AG-CN2-0020) and mirror plot is provided in figure S3A. Elaiophilin, for which none of the software was able to provide annotation, was manually annotated by comparison with published data** and experimental MS/MS from GNPS public database. Mirror plot is provided in figure S4A.   *Simone M, Iorio M, Monciardini P, Santini M, Cantù N, Tocchetti A, Serina S, Brunati C, Vernay T, Gentile A, Aracne M, Cozzi M, van der Hooft J, Sosio M, Donadio S, Maffioli SI. “The molecules Gateway: A Homogeneous, Searchable Database of 150k Annotated Molecules from Actinomycetes” Journal of Natural Products, 2024,  87, 11, 2615–2628, https://doi.org/10.1021/acs.jnatprod.4c00857 **(biblio ref already present)   Comment: In the discussion section, the reference to "purified molecules" when describing the activity of elaiophylin and nigericin is inaccurate, as the compounds were synthesised rather than purified from crude extracts. Therefore, it would be more accurate to refer to them as "pure compounds".

**Authors response**: Thank you for your comment. We agree. The pure compounds were then purchased. We revised the manuscript accordingly in (page 6 line 266) and (page 16 line 576).   Comment: Finally, the limitations of the screening strategy need to be discussed in more depth. In particular, the use of mass spectrometry for structural characterisation. The identified compounds (elaiophylin and nigericin) contain a large number of chiral centres; however, mass spectrometry is stereochemically blind, which raises some doubts about the true identity of the metabolites present in the crude extracts. Authors response: We thank the reviewer for highlighting the stereochemical limitations of mass spectrometry. Indeed, MS/MS alone does not provide direct stereochemical information. However, in our study, metabolite identification was supported by multiple complementary criteria: high-resolution accurate mass measurements, detailed fragmentation patterns, retention time alignment, and crucially, the matching of daughter ion spectra with those reported in the literature for known compounds like elaiophylin and nigericin. These combined pieces of evidence offer a robust and reliable annotation of the metabolites in our extracts. While techniques such as NMR or chiral chromatography are necessary for full stereochemical resolution and discovery of novel stereoisomers, the MS-based dereplication approach used here is well accepted for identifying known natural products and suffices to support the conclusions of our screening platform. We clarified this point in the manuscript to accurately convey both the strengths and limitations of our identification strategy (page 14 line 467-472).  

**Minor points: Comment:** Using the plate position instead of the Extract ID in the data plotting can be confusing for the reader.

**Authors response:** Thank you for the suggestion. However, we think that the name of the extract would be too long to read on the plot, in addition, they are provided in the supplementary table.

**Comment: **Interchanging the strain colour in panels A and B of Figure 5 makes interpretation of the results difficult. **Authors response** This has now been changed. **Comment**: Depositing the mass spectrometry data in a repository like MassIVE would greatly benefit open science. However, the context of a private collection is understandable. **Authors response:** Thank you.
